# BK ablation attenuates osteoblast bone formation via integrin pathway

**DOI:** 10.1038/s41419-019-1972-8

**Published:** 2019-09-30

**Authors:** Yinhang Wang, Qiang Guo, Hongya Hei, Jie Tao, Yi Zhou, Jibin Dong, Hong Xin, Hui Cai, Jianjun Gao, Ker Yu, Svetlana Reilly, Peihao Yin, Xuemei Zhang

**Affiliations:** 1 Department of Pharmacology, School of Pharmacy, Fudan University, Shanghai, 201203 China; 2 Department of Bone Metabolism, Institute of Radiation Medicine, Fudan University, Shanghai, 200032 China; 3 Department of Nephrology, Putuo Hospital, Shanghai University of Traditional Chinese Medicine, Shanghai, 200062 China; 4 Department of Medicine, Renal Division, Emory University School of Medicine, Atlanta, GA 30322 USA; 5 Section of Nephrology, Atlanta Veteran Administration Medical Center, Decatur, GA 30033 USA; 6 Division of Cardiovascular Medicine, Radcliffe Department of Medicine, University of Oxford, John Radcliffe Hospital, Oxford, OX3 9DU UK

**Keywords:** Bone, Bone quality and biomechanics

## Abstract

Impaired bone formation is one of the major causes of low bone mass and skeletal fragility that occurs in osteoporosis. However, the mechanisms underlying the defects in bone formation are not well understood. Here, we report that big conductance calcium-activated potassium channels (BKs) are required for bone formation and osteoblast function both in vivo and in vitro. By 15 weeks of age, BK knockout (BKO) mice exhibited a decline in bone mineral density and trabecular bone volume of the tibiae and lumbar vertebrae, which were associated with impaired bone formation and osteoblast activity. Mechanistically, BK ablation in bone and bone marrow mesenchymal stem cells (BMSCs) of BKO mice inhibited integrin signaling. Furthermore, the binding of α subunit of BK with integrin β1 protein in osteoblasts was confirmed, and FAK-ERK1/2 signaling was proved to be involved by genetic modification of KCNMA1 (which encodes the α subunit of BK) in ROS17/2.8 osteoblast cells. These findings indicated that BK regulates bone formation by promoting osteoblast differentiation via integrin pathway, which provided novel insight into ion transporter crosstalk with the extracellular matrix in osteoblast regulation and revealed a new potential strategy for intervention in correcting bone formation defects.

## Introduction

Defects in bone formation in response to the accelerated bone resorption cause an imbalance in bone remodeling resulting in low bone mass and skeletal fragility in osteoporosis^1,2^. These defects are characterized by a decrease in cell number and function of osteoblasts that are thought to be related to a decline in growth factors (e.g., insulin-like growth factor-1, IGF-1), a deficiency of estrogen and androgen, an accumulation of tumor necrosis factor alpha (TNFα) or ageing;^3–5^ the detailed mechanisms underlying changes in osteoblast functional responses are not understood.

Recently, one of the subtypes of potassium (K^+^) channels, the big conductance calcium-activated potassium channel (BK, also named Maxi K or KCa1.1), has gained considerable interest in bone remodeling^6^, as global deletion of BK channels in mice caused osteopenia due to enhanced osteoclast resorption secondary to the autonomous release of cathepsin K (one of the major enzymes in the degradation of bone matrix)^6^. Potassium channels are a diverse family of membrane proteins that include calcium (Ca^2+^)-activated potassium channels (termed Kca), voltage-gated potassium (K^+^) channels (termed Kv) and others (K2p and Kir channels)^7,8^. It has been shown that the activation of Kca channels increases the efflux of K^+^ and limits Ca^2+^ influx coherently through the deactivation of voltage-gated Ca^2+^ channels, which are suggested to be involved in cell signal transduction and viability in excitable cells, such as neurons, cardiomyocytes and smooth muscle cells and in the regulation of K^+^ homeostasis or cell volume in non-excitable cells, such as tumor cells, bone cells and kidney epithelium^9^. The Kca channels are subdivided into three types according to their conductance: big conductance Kca (BK) channels, intermediate conductance (IK) channels and small conductance (SK) channels^7^. The BK channel consists of four pore-forming α-subunits and several regulatory β or γ subunits^10–12^. The uniqueness of the channel is that it can be activated by both changes in voltage and intracellular Ca^2+^ concentration, which link intracellular Ca^2+^ with membrane electrical signaling^10^. In neurons, heart and bladders, BK channels participate in vascular contractility regulation, neurotransmitter release and hormone secretion^10,11^. Recent reports in non-excitable cells, including bone cells, suggest BK channels regulate cellular functions through intracellular biochemical mechanisms rather than electrical signaling^12,13^.

BK channels are expressed in primary osteoblasts^14–16^, osteoblast cell lines (e.g., MG63, SaOS2, and ROS17/2, 8)^14,16–21^ and progenitors (e.g., mesenchymal stem cells)^22–24^. Emerging evidence indicate that the BK channels in osteoblasts may act as mechanosensors to transduce extracellular mechanical signals into cell; Davidson et al. was the first to report activation of BK channels in human G292 osteoblast-like osteosarcoma cells by the membrane stretch;^19,25^ in human ondonosteoblasts, BK channels also exhibit mechanosensitivity^26^.

The focal adhesion kinase (FAK) is an important player in mechanotransduction; recent studies show that the interaction of FAK with the C-terminus of BK channels is markedly enhanced by hypotonic shock-induced cellular deformation in human osteoblasts^16,27^. BK channels were also found in the activation of tyrosine phosphorylated prtein Syk by PGE2. FAK and Syk tyrosine kinase belong to the family of cytosolic tyrosine kinases and involve in cytoskeleton organization and signal transduction mediated by integrins^28–31^. Induction of integrin β1 leads to FAK aggregation and phosphorylation (auto-phosphorylation at Y397 site) activates MAPK and ERK kinases, and transcription factors-Runx2 and Osterix^32–35^. Runx2, in turn, triggers a cascade of osteoblast markers, including osteopontin (OPN), collagen I, alkaline phosphatase (ALP) and osteocalcin (OCN), to promote the differentiation of osteoblasts for bone matrix synthesis and mineralization^32,35^.

The BK channels are reported to affect cell proliferation and differentiation; for instance, BK channel blockers, TEA and tetrandrine, cause significant changes MG63 cell numbers;^16,27^ in the same cell type secretion of OCN is modulated by BK^17^. Similarly, silencing BK in human BMSCs decrease osteogenic differentiation by reducing mineral precipitation and OCN^24^. We have previously observed that BK knockout in ROS17/2.8 osteoblast cells posses a lower expression of runt-related transcription factor 2 (Runx2, the core transcription factor in osteoblast differentiation) associated with a reduction in osteoblast markers OPN and OCN^21^. Activity of the BK channels can be regulated by prostaglandin E2 (PGE2)^18,25,36^, via the recruitment and activation of tyrosine phosphorylated protein Syk. These channels can also be activated by parathyroid hormone (PTH)^18^, likely by the triggered increase in intracellular Ca^2+^ concentration and estrogens^37,38^ that are major modulators of bone remodeling^39^. Moreover, the expression of BK is downregulated in the long bones of the osteoporosis model induced by ovariectomy (Supplemental Fig. 1). These findings suggest that BK channels might play a role in the regulation of bone formation under physiological and pathological conditions.

To test this hypothesis, we generated BK knockout mice using the CRISPR-Cas9 system and examined the bone formation index (indices) both in vivo and in vitro. The expression of KCNMA1 (which encodes the α subunit of BK) was further modified (silenced or overexpressed) in osteoblast cell lines, and the role of the integrin pathway in BK signaling was explored.

## Results

### Bone loss in BKO mice

**Fig. 1:**
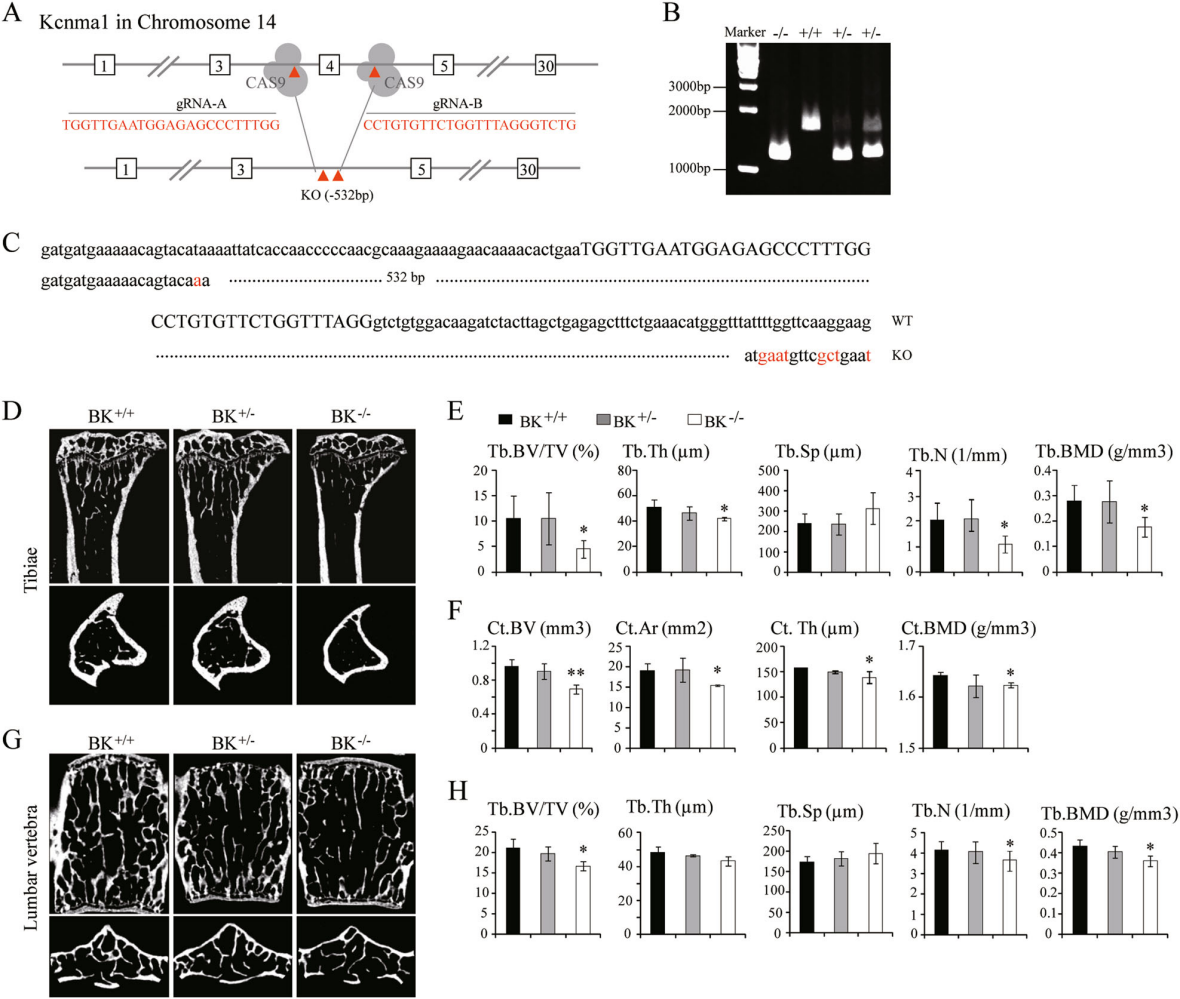
Bone loss in BK channel knockout (BKO) mice. **a** Schematic outlining the generation of BK knockout mice using the CRISPR/Cas9 system. The targeting sites of KCNMA1 (gene encoding the α subunit of BK, BKα) are shown. **b**, **c** BKO female mice were established by breeding BK^+/−^ males and females. The targeted fragment of KCNMA1 was amplified by PCR using genomic DNA templates, and the BK channel deletion was confirmed by sequencing. **d**–**h** Trabecular and cortical bone loss are shown in the tibiae and lumbar vertebrae of BKO female mice at 15 weeks of age. **d** MicroCT images of the proximal tibiae. **e** The trabecular bone volume/tissue volume (Tb.BV/TV), trabecular thickness (Tb.Th), trabecular separation (Tb.Sp), trabecular number (Tb.N) and bone mineral density (Tb.BMD) of the proximal tibiae. **f** The cortical bone volume (Ct.BV) intersection surface (Ct.Ar), thickness (Ct.Th) and bone mineral density (Ct.BMD) of the tibiae. **g** MicroCT images of the coronal plane (upper) and transverse section (below) of the 2nd lumbar vertebrae. **h** The trabecular bone volume/tissue volume (Tb.BV/TV), trabecular thickness (Tb.Th), trabecular separation (Tb.Sp), trabecular number (Tb.N) and bone mineral density (Tb.BMD) of the lumbar vertebrae. Values shown are shown as the mean ± SD, *n* = 3, versus BK^+/+^: *P < 0.05, **P < 0.01

BK channel knockout mice (BKO) were generated by the deletion of exon 4 of KCNMA1 (which encode the pore-forming α-subunits of BK) using the CRISPR/Cas9 strategy. (Fig. 1a). The BKO female mice, which carried a 532-bp fragment deletion from 23591392 to 23591923 bp in the KCNMA1 genome DNA sequence (NC 000080.6), was identified by PCR (Fig. 1b) and confirmed by sequencing (Fig. 1c) and whole cell patch clamp study (Supplemental Fig. 2).

The significant reduction of trabecular bone in the tibiae and lumbar vertebrae was revealed by microcomputed tomography (μCT) analysis in the BKO female mice at 15 weeks of age (Fig. 1d, g). The trabecular bone volume/tissue volume (Tb. BV/TV) in the tibia of BKO mice was markedly decreased by 56.80% (*P* < 0.05) compared with that of their wild-type (WT) littermates; the trabecular thickness (Tb.Th) was decreased by 17.83% (*P* < 0.05), and the trabecular number (Tb.N) was decreased by 47.50% (*P* < 0.05), whereas trabecular separation (Tb.Sp) was increased by 130.68% (*P* = 0.06) (Fig. 1e). Additionally, the cortical bone volume (Ct.BV) of the proximal tibiae in BKO mice was decreased by 27.75% (*P* < 0.01) compared with that of their WT littermates, along with decreases in intersection surface area (Ct.Ar) and thickness (Ct.Th) (Fig. 1f). These differences were greater in the long bone (tibia) than in vertebral (lumbar) bone. The BKO mice showed a significant decrease in the trabecular bone mineral density (Tb.BMD) of the proximal tibia and lumbar vertebrae by 36.6% (*P* < 0.05) and 17.4% (*P* < 0.05), respectively (Fig. 1e, h).

### Defects in osteoblast bone formation

**Fig. 2:**
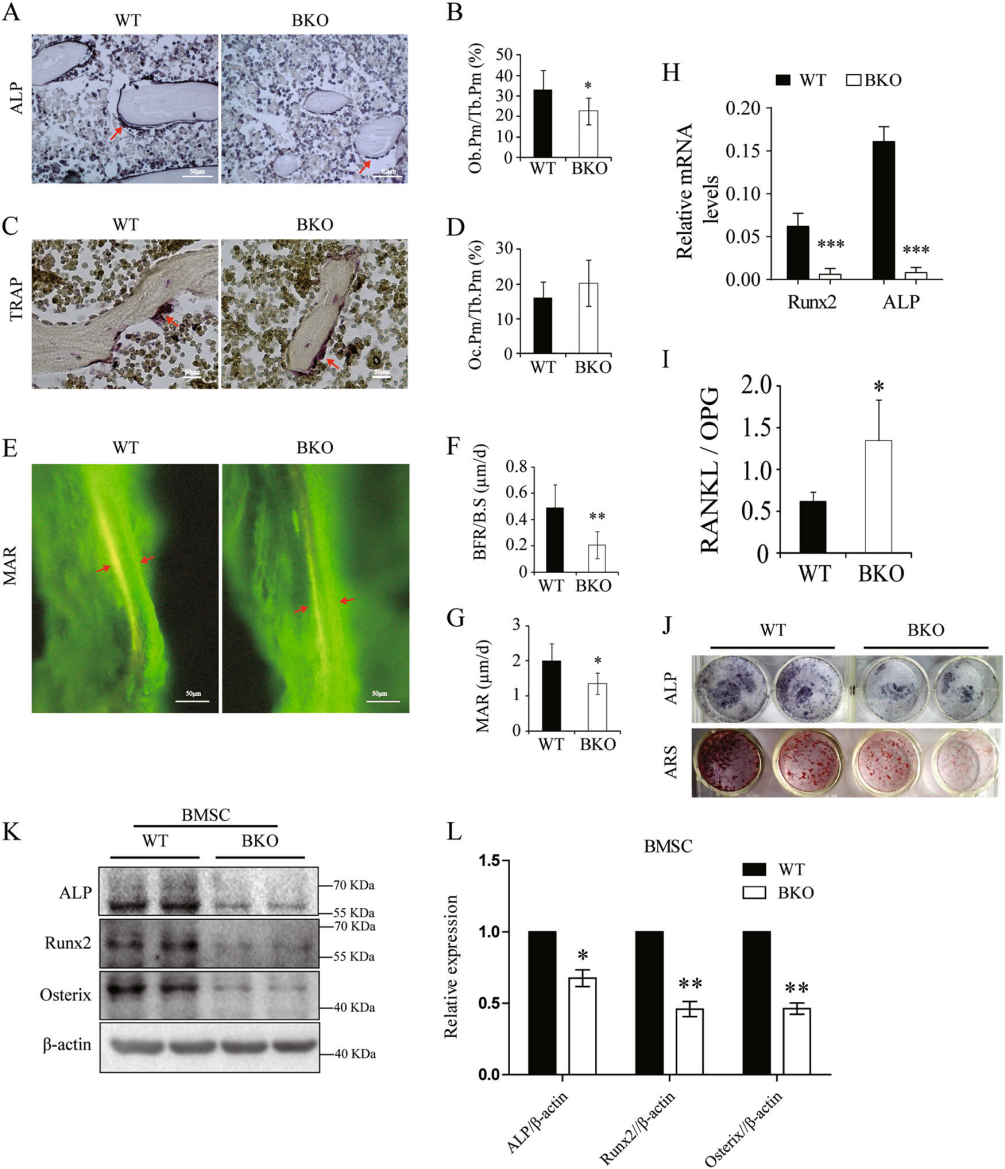
Defects in osteoblast bone formation in BKO mice. **a** ALP staining (scale bar = 50 μm) in the trabecular region of the lumbar vertebrae, and the red arrows pointed to the typical osteoblasts. **b** The osteoblast perimeter (Ob.Pm/Th.Pm) in the trabecular region of the lumbar vertebrae. **c** TRAP staining (scale bar = 50 μm) in the trabecular region of the lumbar vertebrae, and the red arrows pointed to the typical osteoclasts. **d** The osteoclast perimeter (Oc.Pm/Th.Pm) in the trabecular region of the lumbar vertebrae. **e** Tetracycline/calcein labeling (the yellow line is tetracycline labeling and the green line is calcein labeling, scale bar = 50 μm), **f** bone formation rate (BFR/B.S) and **g** mineral apposition rate (MAR) in the trabecular region of the tibiae. Defects in bone formation are shown in BKO mice. Values are shown as the mean ± SD, *n* = 6, versus WT: *P < 0.05, **P < 0.01. **h**, **i** Gene expression of Runx2 and ALP and the ratio of RANKL/OPG were determined in the femurs of BKO and WT mice by real-time RT-PCR. Values are shown as the mean ± SD, *n* = 4, versus WT: **P < 0.01, ***P < 0.005. **j** ALP and Alizarin Red staining (ARS) of BMSCs after 14 and 21 days of culture, respectively, in DMEM supplemented with 50 µg/ml ascorbic acid and 10 mM ß-glycerophosphate. **k**, **l** Expression and quantitative analysis of ALP, Runx2, and osterix of BMSCs after 21 days of culture in DMEM supplemented with 50 µg/ml ascorbic acid and 10 mM ß-glycerophosphate. Values are shown as the mean ± SD, *n* = 6, versus WT: *P < 0.05, **P < 0.01

Using histomorphometry, we found that BKO mice have decreased by 31% osteoblast activity (Fig. 2a, b) shown by the osteoblast perimeter per trabecular perimeter (Ob.Pm/Tb.Pm), compared with that in WT mice; by contrast, there was no significant difference in osteoclast activity, as shown by the osteoclast perimeter per trabecular perimeter (Oc.Pm/Tb.Pm) in Fig. 2c, d. The bone formation rate (BFR/B.S) and the mineral apposition rate (MAR) were decreased by 57.8% (*P* < 0.01) and 12.5% (*P* < 0.01), respectively (Fig. 2e–g). Furthermore, the gene expression levels of Runt-related transcription factor 2 (Runx2) and the osteoblast marker ALP were significantly decreased in the bones of BKO mice as determined by real-time RT-PCR (Fig. 2h). The ratio of the gene expression of receptor activator of nuclear factor kappa B ligand (RANKL), the major activator of osteoclasts, to osteoprotegerin (OPG), which is a decoy receptor for RANKL, was modestly increased (Fig. 2i).

The decreases in osteoblast function in BKO mice were further verified by decreases in alkaline phosphatase (ALP) activity and its ability to form mineralized nodules, as determined by ALP and Alizarin Red staining, respectively, in bone marrow stromal cell (BMSC) cultures (Fig. 2j). Consistently, the expression levels of Runx2, Osterix, and ALP were significantly decreased in BK-deficient BMSCs (Fig. 2k, l). Taken together, these data indicate that BK channel ablation inhibits the osteoblast differentiation and bone formation.

### FAK/ERK involved in BK action in osteoblasts

**Fig. 3:**
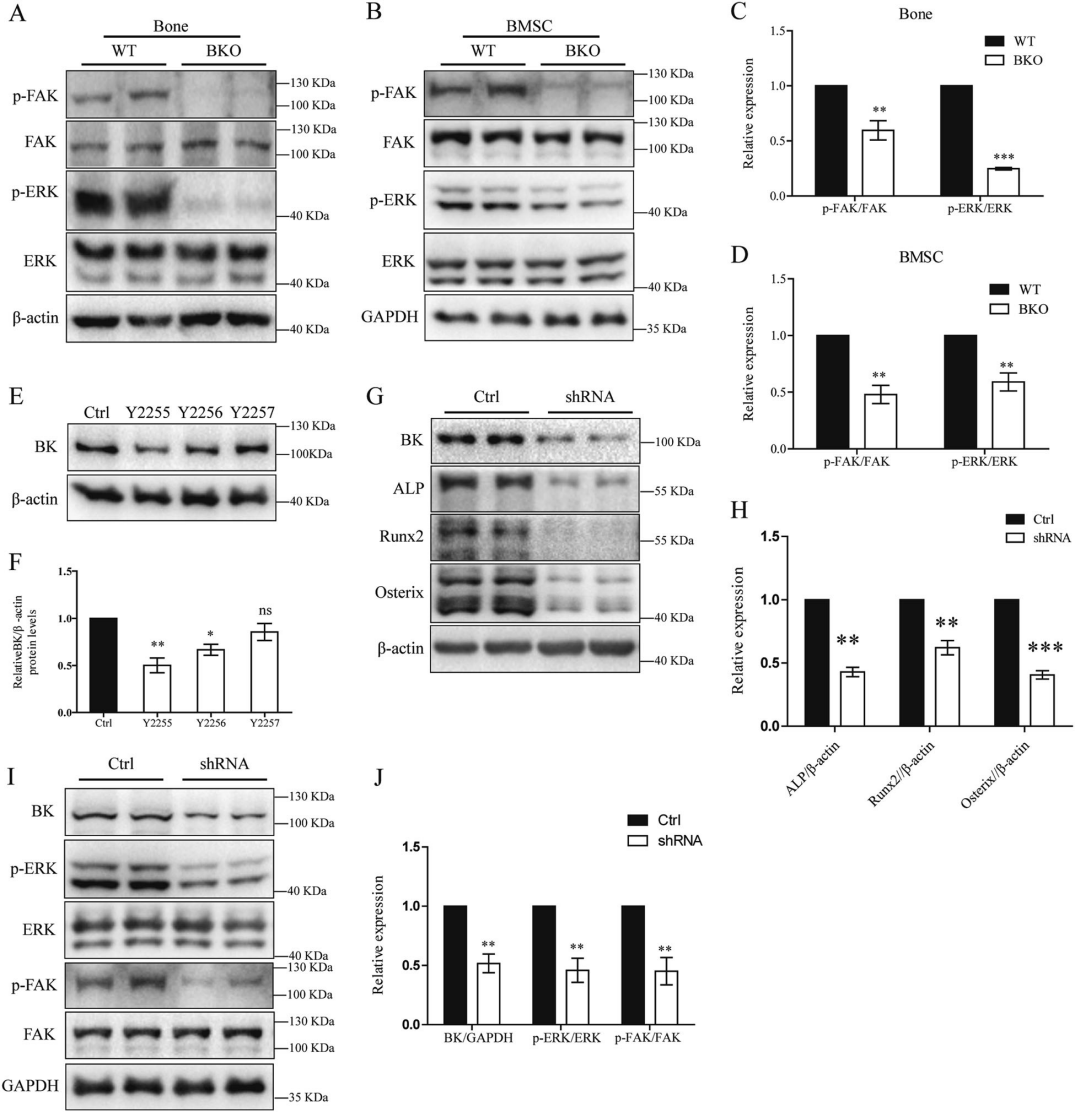
Decrease in signaling of FAK/ERK by BK knockdown. **a** Phosphorylated levels of p-FAK-Y397 and p-ERK1/2 in the bone of BKO mice as determined by western blot analysis. **b** Phosphorylated levels of p-FAK-Y397 and p-ERK1/2 in the BMSCs after 21 days of culture as determined by western blot analysis. **c**, **d** Quantitative analysis of the protein expression levels in bone and BMSCs. Values are shown as the mean ± SD, *n* = 3, versus WT: **P < 0.01, ***P < 0.005. **e**, **f** Expression and quantitative analysis of BKα protein levels by western blot analysis in ROS17/2.8 cells after BK-shRNA transfection. Values are shown as the mean ± SD, *n* = 3, versus Ctrl: *P < 0.05, **P < 0.01, ns, no statistical significance. **g**, **h** Expression and quantitative analysis of BK, ALP, Runx2, and osterix in Y2255-transfected (BK-silenced) ROS17/2.8 cells. Values are shown as the mean ± SD, *n* = 3, versus Ctrl: **P < 0.01, ***P < 0.005. **i**, **j** Expression and quantitative analysis of BK, p-FAK-Y397, and p-ERK1/2 in Y2255-transfected (BK-silenced) ROS17/2.8 cells. Values are shown as the mean ± SD, *n* = 3, versus Ctrl: **P < 0.01

Interestingly, along with the impaired osteoblast differentiation, BK ablation caused a decrease in the phosphorylation of focal adhesion kinase (p-FAK-Y397) and p-ERK1/2 in the bone and BMSCs of BKO mice as determined by western blot analysis (Fig. 3a–d).

To explore the underlying molecular events, the expression of KCNMA1 was further modified in ROS17/2.8 osteoblast cells. Three BKα-shRNA plasmids Y2255, Y2256, and Y2257 were transfected into ROS17/2.8 cells. As shown in Fig. 3e, f, BKα was knocked down at the protein level by Y2255 plasmid. In line with findings in BKO mice, silencing BK expression caused a significant decrease in Runx2, osterix, and ALP (Fig. 3g, h), along with p-FAK-Y397 and p-ERK1/2 (Fig. 3i, j), which are two major mediators of signal transduction via integrins. Similar results are also found in MC3T3-E1 osteoblasts after BK knocking down (Supplemental Fig. 3a, b).

**Fig. 4:**
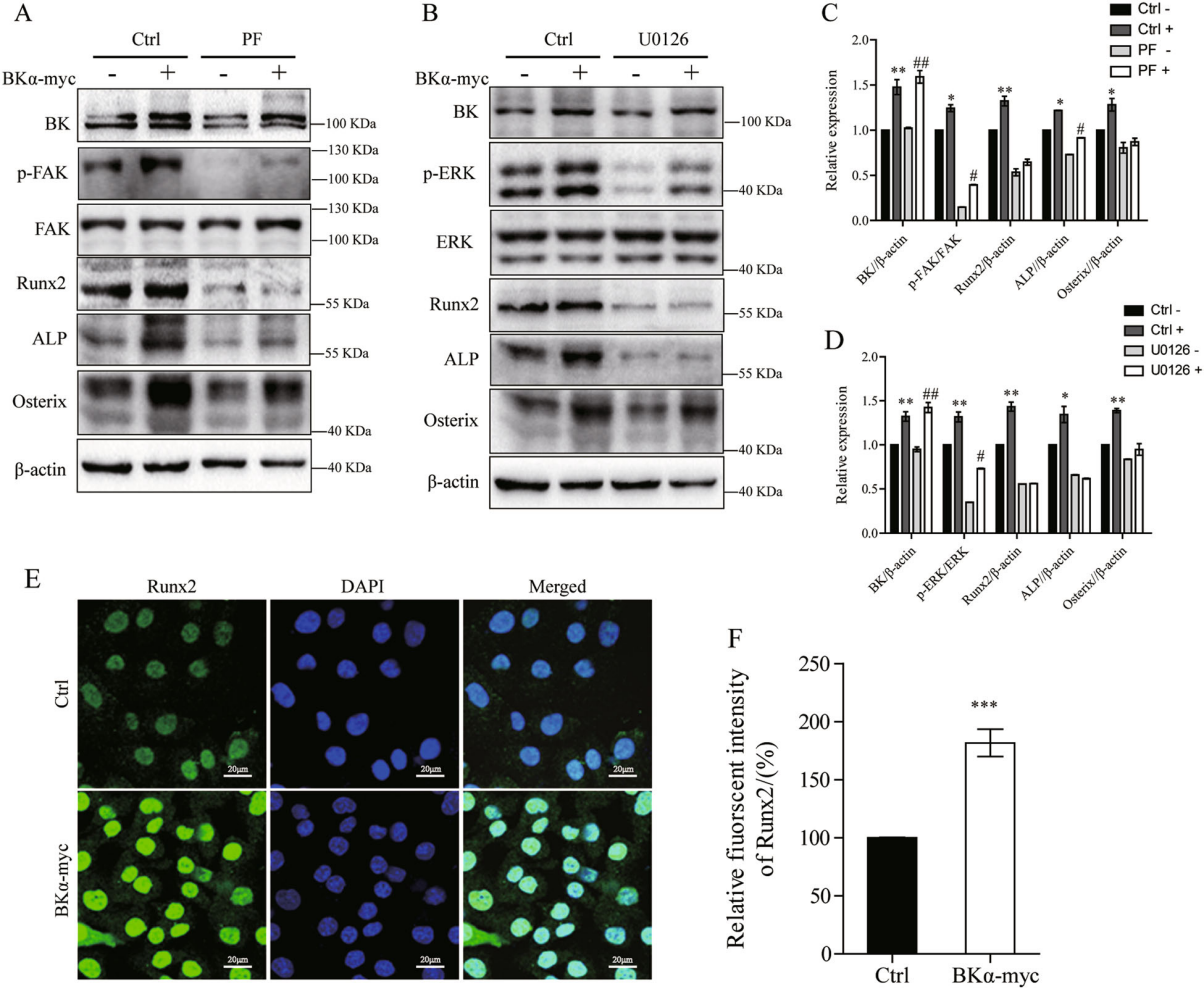
Overexpression of BK increases osteogenic markers through FAK and ERK. **a** Western blot analysis of BK, p-FAK-Y397, Runx2, ALP, and Osterix after the BKα-myc plasmid was transfected into ROS17/2.8 cells using the FAK inhibitor PF-562271 (5 μM). PF was short for PF-562271. **b** Western blot analysis of BK, p-ERK1/2, Runx2, ALP, and osterix in BKα-myc plasmid transfected ROS17/2.8 cells using the ERK inhibitor U0126 (10 μM). **c**, **d** Quantitative analysis of the protein expression levels by western blot of (**a**, **b**). Values are shown as the mean ± SD, *n* = 3, versus Ctrl -: *P < 0.05, **P < 0.01, versus PF- or U0126 -: ^#^*P* < 0.05, ^##^*P* < 0.01. **e** Immunofluorescent staining of Runx2 in BKα-myc transfected ROS17/2.8 cells. Green is FITC-stained Runx2, and blue is DAPI-stained nuclei. **f** Quantification of Runx2 expression by immunofluorescence. Values are shown as the mean ± SD, *n* = 3, versus Ctrl: ***P < 0.001

To further explore the BK actions in osteoblasts, we next overexpressed BK channels using the BKα-myc plasmid in ROS17/2.8 cells (Fig. 4a–d) and MC3T3-E1 cells (Supplemental Fig. 4a–d). As shown in the results, overexpression of BK led to the increased protein level of Runx2 that was blocked by the FAK inhibitor PF-562271(Fig. 4a, c) or the ERK1/2 inhibitor U0126 (Fig. 4b, d). The increase in Runx2 in the nucleus of osteoblasts was confirmed by immunofluorescent staining (Fig. 4e, f). Similar results were observed in MC3T3-E1 cells (Supplemental Fig. 4a–d). These results indicate that the FAK-ERK1/2 signaling pathway is involved in the activation of Runx2 by BK in osteoblasts.

### Interaction between BK and integrin β1 in osteoblasts

**Fig. 5:**
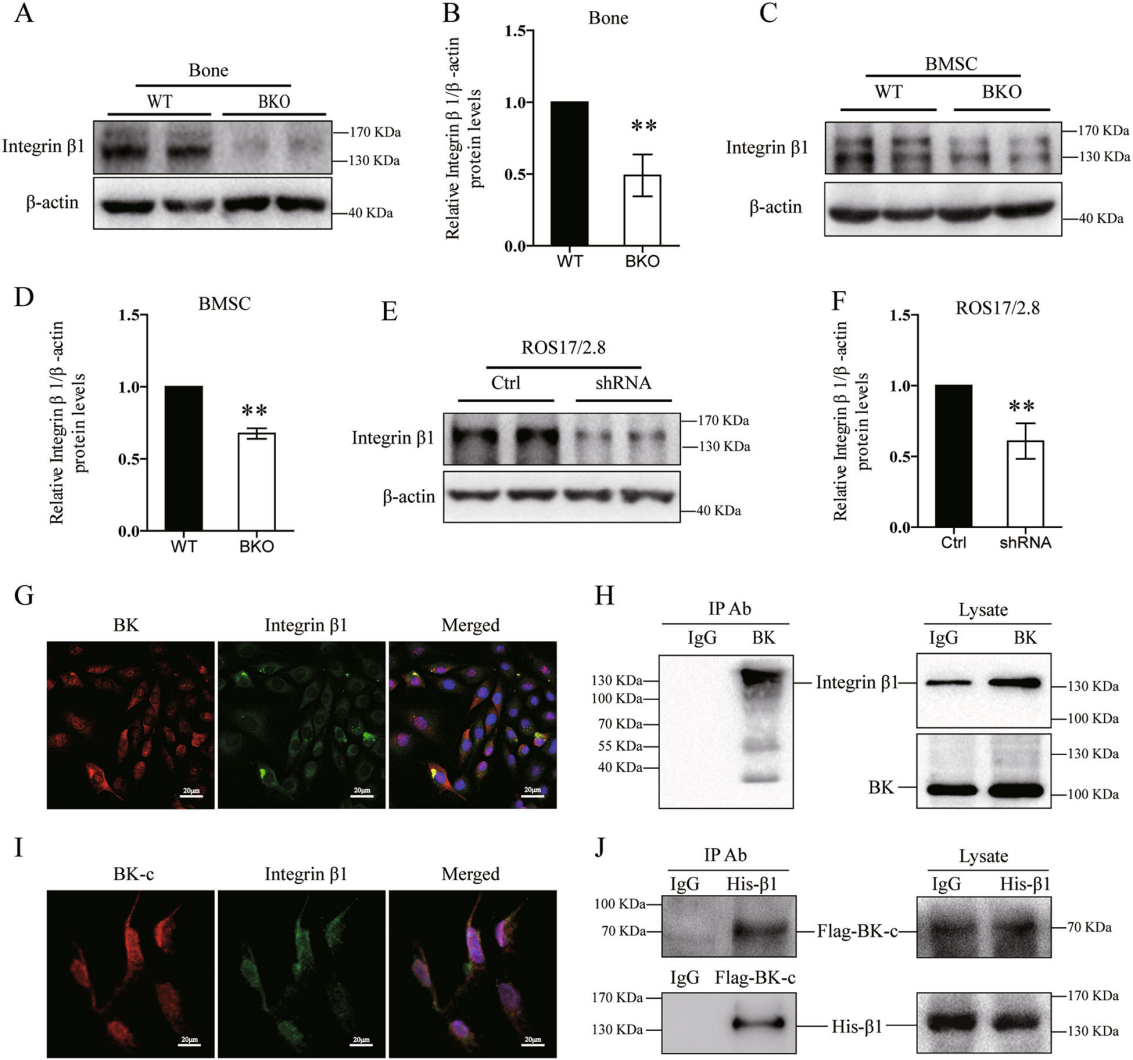
The interaction of BK with integrin beta1 in osteoblasts. **a**, **b** Expression of integrin β1in the bone of BKO mice as determined by western blot analysis. Values are shown as the mean ± SD, *n* = 3, versus WT: **P < 0.01. **c**, **d** Expression of integrin β1 in the BMSCs after 21 days of culture in vitro as determined by western blot analysis. Values are shown as the mean ± SD, *n* = 3, versus WT: **P < 0.01. **e**, **f** Expression of integrin β1 in the BK-silenced ROS17/2.8 cells as determined by western blot analysis. Values are shown as the mean ± SD, *n* = 3, versus Ctrl: **P < 0.01. **g** Immunofluorescent staining of BKα and integrin β1 in ROS17/2.8 osteoblasts. BK protein was stained by Rhodamine red X (shown in red), and integrin β1 protein was stained by FITC (shown in green). Both proteins have almost the same localization at the plasma membrane of osteoblasts. **h** BKα protein was overexpressed in ROS17/2.8 cells. Co-immunoprecipitation (Co-IP) was performed by immunoprecipitation (IP) using the anti-BK antibody, and western blotting was performed using anti-integrinβ1 antibodies. **i** A BK-C-Flag plasmid (encoding 380–1243 amino acids, including the C-terminal region of BKα) and the integrin β1-His plasmid were co-transfected into HEK293T cells. BK-C was stained by Rhodamine red X (shown in red) and integrin β1 protein was stained by FITC (shown in green) using anti-Flag and anti-his antibodies, respectively, in immunofluorescent staining. **j** The Co-IP was performed using anti-Flag and anti-His antibodies, and integrin β1 and BK-C were identified by western blot analysis

Genetic deletion of the BK in mice showed reduced levels of integrin β1 in bone and BMSCs (Fig. 5a–d). Similar the expression levels of integrin β1 were decreased in the BK knockdown ROS17/2.8 (Fig. 5e, f) and MG63 cells (Supplemental Fig. 5a), while BK overexpressing MG63 cells had increased integrin β1 (Supplemental Fig. 5b). To test whether integrin β1 regulates BK expression, we knockdown integrin β1with the integrin β1-shRNA plasmid. The results showed that downregulation of integrin β1 did not affect the expression of BK (Supplemental Fig. 5c, d). Thus BK is likely to be located upstream of integrin β1 signaling in osteoblasts.

To determine the interaction of BK with integrin β1 in osteoblasts, immunocytochemical staining (ICC) and co-immunoprecipitation (Co-IP) were performed in transfected BKα-myc ROS17/2.8 cells. As shown in Fig. 5g, BKα and integrin β1 have almost the same localization according to fluorescent staining; the integrin β1 protein was also detectable in the BK immunoprecipitates (Fig. 5h).

To explore the binding site between BK and integrinβ1, we co-transfected HEK293T cells with the BK-C-Flag plasmid (encoding 380–1243 amino acids, including the C-terminal region of BKα) and the integrin β1-His plasmid. Figure 5i shows that BK-C and integrin β1 co-localize that was also observed in Co-IP experiments using anti-Flag antibody and anti-His antibody (Fig. 5j). These results indicate that BK (preferentially at the C-terminus of BKα) binding to integrin β1 (probably to stabilize the protein) activates Runx2 and osteoblast differentiation through FAK-ERK1/2 signaling pathway.

## Discussion

In the present study, we generated a BK knockout mouse by the deletion of exon 4 of KCNMA1 (the gene encoding the pore-forming α-subunit of BK, BKα) using the CRISPR/Cas9 strategy. By 15 weeks of age, a significant reduction in the trabecular and cortical bone of BK-deficient (BKO) female mice was revealed by μCT analysis. Importantly, defects in bone formation in these BKO mice were detected by histomorphometric analysis. The osteoblast activities, which were evaluated by osteoblast perimeter (Ob.Pm/Tb.Pm), mineral apposition rate (MAR) and bone formation rate (BFR/B.S) were decreased in BKO mice, with no or modest changes in osteoclast activity. The impaired osteoblast function was further identified by decreases in ALP activity and its ability to form mineralized nodules in BMSC cultures. This finding was further confirmed by the reduction of Runx2 and ALP expression in the bones and BMSCs of BKO mice. These results demonstrated that the low bone mass characterized by BK deficiency was mostly, if not completely, due to a defect in bone formation from impaired osteoblast activity and osteoblast differentiation.

Osteoblasts are bone-forming cells responsible for bone matrix synthesis and its subsequent mineralization^39^. The cells arise from the differentiation of mesenchymal stem cells (MSCs) under the control of local (factors or signals from extracellular matrix) and systemic (e.g., PTH, 1α, 25[OH]_2_D) factors^40^. Runt-related transcription factor 2 (Runx2) is the master transcription factor in this process^32,33,41,42^ and could be activated via signaling pathways, such as the mitogen-activated protein kinase (MAPK) pathway or the extracellular signal-regulated kinase (ERK) pathway^33,34^. The activation of Runx2 then triggers a cascade of the downstream osteoblast markers OPN, collagen I, ALP and OCN to promote the differentiation of osteoblasts for bone matrix synthesis and mineralization^35^. The decrease in the phosphorylation of focal adhesion kinase (p-FAK-Y397) and p-ERK1/2 found in bone and BMSCs of BKO mice, as well as the reduced protein level of integrin β1, indicated altered integrin signaling. To explore the role of integrin signaling in osteoblast defects by BK deficiency, the BK pore forming α-subunit gene KCNMA1 were further modified in ROS17/2.8 and MC3T3-E1 osteoblasts (silenced by BKα-shRNA and overexpressed with the BKα-myc plasmid), and the expression of Runx2 and the signaling pathway were determined. In line with the findings in BKO mice and BKO BMSCs, silencing BK caused a decrease in Runx2 and Osterix and the downstream osteoblast markers ALP; these were paralleled with a decrease in the phosphorylation of focal adhesion kinase (p-FAK-Y397) and p-ERK1/2. Correspondently, overexpressing BK induced Runx2, osterix, and ALP, which could be abolished by inhibitors of FAK or ERK1/2. These results indicate that Runx2, acting as one of the key targets, could be activated by BK through the FAK/ERK pathway in osteoblasts.

Integrins are a family of transmembrane α/β heterodimer adhesion molecules that convey signals to (outside-in signaling) and from (inside-out signaling) the cytosol across the plasma membrane^31,43^. Integrins, especially integrin β1, play important roles in bone formation. The deletion of β1 or its regulatory factor ICAP-1 decreases osteoblast differentiation and bone formation, leading to a significant decrease in bone mineral density^44,45^. The activation of integrin β1 leads to FAK aggregation and phosphorylation (autophosphorylation at Y397 site), thereby triggering a series of downstream signaling pathways, such as MAPK/ERK1/2, and further activating Runx2 to promote osteoblasts differentiation^33–35,46^. Integrin β1 decreased by BK deficiency in the bone and BMSCs of our BKO mice and in BKα-silenced osteoblasts. To further explore the relationship of BK and integrin β1, the interaction of these two molecules was evaluated in KCNMA1- or integrin β1-modified cells. As expected, the binding of full-length BKα with integrin β1 was confirmed by immunocytochemistry and Co-IP. Although integrin β1 is decreased by BK silencing, the downregulation of integrin β1 did not affect the expression of BK (supplemental data), indicating that BK acts upstream of integrin β1 signaling in osteoblasts. These results suggest that integrin β1 mediates the actions of BK in osteoblasts.

The BK α-subunit consists of a transmembrane domain (including seven transmembrane segments), a short extracellular N terminus and a large intracellular C-terminus. The regulator of conduction of K^+^ (RCK) in the intracellular region of the C-terminus of BK is the main region for the phosphorylation of BK^47–49^. To determine its binding site, the C-terminus of BKα (380–1243 amino acid, including RCK regions) and integrin β1 were overexpressed in HEK293T cells by the co-transfection of a BK-C-Flag plasmid and integrin β1-His plasmid. The co-expression of BK-C and integrin β1 was confirmed by immunocytochemistry and Co-IP experiments. These observations indicate that the C-terminus of BKα binds to integrin β1 (probably to stabilize the protein) and promotes osteoblast differentiation through the activation of FAK and ERK1/2.

**Fig. 6:**
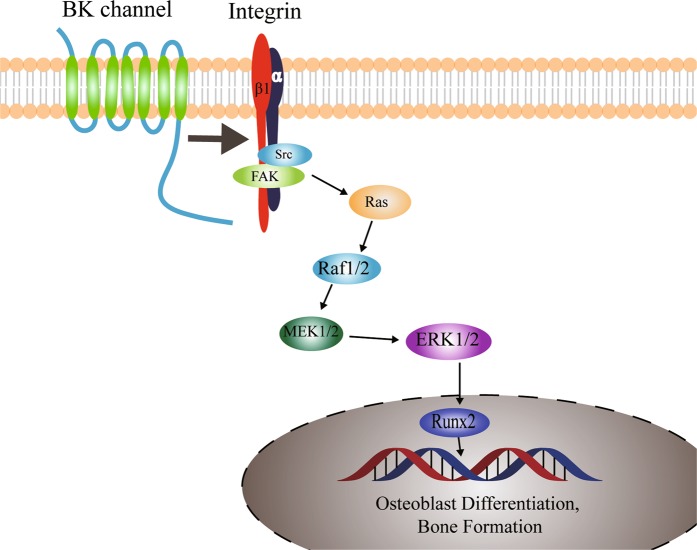
The illustration of BK action in osteoblasts. BK protein (preferentially via C-terminus of BKα) binds integrin β1 in osteoblasts. By binding to integrin β1 (probably to stabilize the protein), BK activates the signaling of integrins, such as FAK and ERK, increases transcription factor Runx2 and accelerate osteoblast differentiation and bone formation

In summary, our studies demonstrated the critical role of BK channels in maintaining skeletal integrity in BK-deficient mice. The reduction in bone mass mostly results from defects in bone formation caused by impaired osteoblast function due to the absence of BK through integrin signaling. There is an interaction between BK protein (especially the C-terminus of BKα) and integrin β1 in osteoblasts. Interaction between BK and integrin β1 activates integrin signaling, via FAK and ERK subsequently leading to the increase in the transcription factor Runx2 expression and accelerated osteoblast differentiation and bone formation (as illustrated in Fig. 6). Our study suggests an important role of BK in maintaining bone formation and in the pathogenesis of osteoporosis. And thus, BK channels may represent a new therapeutic target for osteoporosis.

## Materials and methods

### Generation of BK knockout mice using the CRISPR/Cas9 strategy

Two pairs of oligonucleotides, (TAGGAATTTCTCTCCATTCAACCA and AAACTGGTTGAATGGAGAGCCCTT; TAGGCAGACCCTAAACCAGAACAC and AAACGTGTTCTGGTTTAGGGTCTG) for gRNA targeting on exon 4 of KCNMA1 (which encodes the pore-forming α subunits of BK) were transcribed in vitro. A mixture of Cas9 mRNA (25 ng/μl) and gRNA (12.5 ng/μl per gRNA) was microinjected into the cytoplasm of zygotes of C57BL/6 mice (Bioray Laboratories Inc, Shanghai, China.). Several types of frame shift mutations were found in pups. One of these mutations was chosen to establish a colony (designated BKO), which carried a 532-bp fragment deletion (including the entire exon 4 of KCNMA1) from 23591392 to 23591923 bp in the genome DNA sequence (NC 000080.6). Genotyping was performed by PCR on DNA isolated from tails using the BK primer sequences 5’-TCTCCATACTCCCTCCCTT-3’ and 5’- CCTAATCCTACAAGCCTTCACT -3’ (a 1531-bp fragment for WT and a 1000-bp fragment for the KCNMA1 knockout gene) and confirmed by sequencing.

The BKO mice were established by breeding BK^+/−^ males and females. These breeding pairs provided ^+/+^ (wild-type), ^+/−^ (heterozygous) and ^−/−^ (BKO) littermates and the female mice were used for a direct comparison. In all experiments, tissues were collected at the time of sacrifice for further study. All mice were maintained in a virus- and parasite-free barrier facility and exposed to a 12-h/12-h light/dark cycle. All experiments involving animals were performed according to institutionally approved and current animal care guidelines.

### Microcomputed tomography (μCT)

Samples of tibiae and the second lumbar vertebra (L2) obtained from female mice at 15 weeks of age were dissected free of soft tissue, fixed overnight in 70% ethanol and analyzed by μCT with a SkyScan 1172 scanner and associated analysis software (SkyScan, Belgium). Scans were performed on the whole L2 and the proximal part of the tibia. Image acquisition was performed at 59 kV and 167 μA, with a 0.6° rotation between frames. Thresholding (85/255) was applied to the images to segment the bone from the background. The 3D reconstruction was performed using 2-dimensional data from scanned slices with the 3D Creator software supplied with the instrument. For lumbar reconstruction, the trabecular bone volume of interest (VOI) was drawn to include all cancellous bone in the whole cavity. For tibia reconstruction, the VOI was drawn to include 100 slices of metaphyseal spongiosa (~1 mm region) starting from 1 mm below the growth plate. The trabecular bone volume/tissue volume (BV/TV), trabecular thickness (Tb.Th), trabecular separation (Tb.Sp) and bone mineral density (BMD) as well as the cortical bone volume (Ct.BV), cortical bone thickness (Ct.Th) and cortical bone intersection surface area (Ct.Ar) were calculated as previously described^50^. The resolution of the μCT images was 9.92 microns.

### Histomorphometry

The femur and vertebrae (L3) were removed and fixed in PLP fixative (2% paraformaldehyde containing 0.075 M lysine and 0.01 M sodium periodate solution) overnight at 4 °C and processed histologically using a modified method^51^. The bones were decalcified in an ethylene-diamine tetra-acetic acid (EDTA) glycerol solution for 2 weeks at 4 °C. Decalcified bones were dehydrated and embedded in paraffin, after which 4 μm sections were cut on a rotary microtome. The sections were stained with Mayer’s hematoxylin and eosin (H&E) and histochemically tested for alkaline phosphatase (ALP) activity using a BCIP/NBT kit (C3206, Beyotime Biotechnology, China) and for tartrate-resistant acid phosphatase (TRAP) activity using a TRACP kit (387-A Leukocyte Acid Phosphatase Kit, Sigma-Aldrich, USA).

After staining, images of fields in the metaphyseal area below the growth plate were viewed and imaged with a microscope (Imager.M2, Zeiss, Germany). Images of micrographs from single sections were digitally recorded using a rectangular template, and the recordings were processed for histomorphometric measurements and analyzed using Image-Pro Plus software (Media Cybernetics, USA). The parameters obtained for bone formation were the ALP-positive osteoblast perimeter per trabecular perimeter (Ob.Pm/Tb.Pm, %). The parameter measured for bone resorption was the TRAP-positive osteoclast perimeter per trabecular perimeter (Oc.Pm/Tb.Pm, %).

### Tetracycline and calcein labeling

Tetracycline (30 mg/kg) and calcein (10 mg/kg) (C-0875, Sigma, USA) were administered by intraperitoneal injection 10 and 3 days prior to sacrifice. The lumbar vertebrae were harvested and fixed overnight in 70% ethanol. The samples were dehydrated for 1 day in 30% sucrose, embedded in OCT (4583, Tissue-Tek, Jap) and then frozen in the vapor phase of liquid nitrogen. Serial sections were cut on a freeze microtome, and the freshly cut surface of each section was viewed and imaged using fluorescence microscopy (E600, Nikon, Jap) with a Digital Camera (DXM1200, Nikon) and attached software. The single labeled perimeter (sL.Pm), double labeled perimeter (dL.Pm), interlabel width (IrL.Wi), and Trabecular perimeter (Tb.Pm) were measured using Image Pro Plus software. The mineral apposition rate (MAR; MAR = interlabel width/7, μm per day) and bone formation rate (BFR/BS; BFR/BS = (sL.Pm /2 + dL.Pm)/Tb.Pm*MAR) were calculated.

### Cell cultures

bone marrow mesenchyma stem cells (BMSCs) were flushed out from the femur and tibia of mice (>2 months old) with DMEM (Gibco, USA) containing 100 IU/ml penicillin, 100 mg/ml streptomycin (Gibco) and 10% fetal bovine serum (FBS, Gibco) and supplemented with 50 µg/ml ascorbic acid, 10 mM ß-glycerophosphate, and 10^−8^ mol/L dexamethasone (differentiation medium). The cells were dispersed by repeated pipetting, and a single-cell suspension was achieved by forcefully expelling the cells through a 22-gauge syringe needle. The cells from two mice were pooled and cultured in one 6- or 24-well plate (Nunc, USA) in differentiation medium. The cultures were maintained for 14 days for ALP staining or 21 days for Alizarin Red staining and western blot analysis. At the end of the culture period, the cells were washed with PBS, fixed with PLP fixative, and stained for ALP using the BCIP/NBT kit (Beyotime Biotechnology, Jiangsu, China) by a standard procedure or stained in 1 mg/ml Alizarin Red solution (Yeasen, Shanghai, China) for 30 min at room temperature as previously described. After washing with distilled water and drying in air, images of the stained plates were taken.

ROS17/2.8 cells, a rat osteoblastic cell line, were a kind gift from Dr. Sandra Guggino (Johns Hopkins University, Baltimore, MD). ROS17/2.8 cells were cultured in Ham’s F-12 nutrient mixture medium (Sigma-Aldrich, USA) containing NaHCO3 and supplemented with 10% FBS, 100 U/ml penicillin and 100 mg/ml streptomycin. HEK293T cells were cultured in Dulbecco’s Modified Eagle Medium (Gibco, USA) supplemented with 10% FBS, 100 U/ml penicillin and 100 mg/ml streptomycin. The cells were cultured in a humidified atmosphere of 95% air and 5% CO_2_ at 37 °C and subcultured every 2–3 days. For the experiments, the cells were trypsinized at approximately 90% confluence and seeded onto 6-well plates at a density of 2.5 × 10^4^/ml.

### RNA interference

A series of small hairpin RNA (shRNA) targeted to mRNA of BKα (KCNMA1; NM-031828) or integrin β1 (ITGB1; NM-017022) were designed and constructed in the lentivirus vector Plko-CMV-G&PR-U6-shRNA (Obio Technology (Shanghai) Co. Ltd, China) according to the manufacturer’s protocol. Three shRNA plasmids targeted to KCNMA1 (Y2255, targeted to GCGGTTTATTGCAGCCAATGA, Y2256 to GCTTAAGCTCCTGATGATAGC, and Y2257 to GCATCTTGGCGTCACTCAACA) and two shRNA plasmids targeted to ITGB1 (Y2652 to CCACAACAGCTGCTTCTAA and Y2653 to GGAGGATTACTTCAGACTT) were obtained. The plasmid Y007 targeted to TTCTCCGAACGTGTCACGT was used as a negative control. The shRNA plasmids were transfected into ROS 17/2.8 cells in 6-well plates using Lipofectamine 3000 (L3000-015, Invitrogen, USA) according to the manufacturer’s protocol. Four μg/ml puromycin was added for selection on the second day of transfection. Two days later, the cell proteins were extracted for analysis by western blotting.

### BK overexpression

The BKα-myc plasmid was transfected into ROS 17/2.8 cells in 6-well plates using Lipofectamine 3000. The medium was refreshed 24 h later, and 10 μM ERK1/2 inhibitor U0126 (S1102, Selleck, USA) or 5 μM FAK inhibitor PF-562271 (S2890, Selleck) was added as needed. The cells were collected for protein analysis 2 days later. The BK-C-Flag plasmid (code for 380–1243 amino acids, including the region of the c-terminus of BKα) and the integrin β1-His plasmid were designed by Vigene Biosciences (Rockville, MD, USA). The BKα-myc plasmid or BK-C-Flag plasmid with integrin β1-His were co-transfected into HEK293T cells in 6-well plates using Lipofectamine 3000. The cells were collected for protein analysis 2 days later.

### Immunofluorescent staining

Immunocytochemistry analysis was performed according to a previous report^21^. The cells were seeded onto slides. At 70% confluence, the cells were fixed in 4% paraformaldehyde in PBS buffer for 15 min. Following washes with PBS, the cells were permeabilized in freshly prepared 0.1% Triton X-100 for 10 min and blocked in 1% bovine serum albumin (BSA) in PBS buffer for 1 h at room temperature. The cells were incubated with primary antibodies directed against either runx2 (1:200, ab76956, Abcam), the α-subunit of BK (1:100, ab99046, Abcam, USA), integrin beta1 (1:100, 1798-1, Epitomics, USA), Flag (1:200, Rabbit Monoclonal Anti-Flag antibody, F2555, Sigma) or His (1:200, SAB1306084, Sigma) at 4 °C overnight. The secondary Alexa Fluor 647-conjugated anti-mouse antibody (Jackson Immuno Research, USA) was used at a 1:200 dilution for 2 h at room temperature protected from light. Following washes with PBS, the cells were stained with DAPI for 5 min. The slides were mounted with SlowFade™ Gold Antifade Mountant (S36936, Invitrogen), and images were captured using a Leica TCS SP5 confocal microscope (Leica, Germany) at room temperature with ×10 and ×40 objective lenses.

### Quantitative real-time PCR

Total RNA was extracted from long bones using Trizol reagent (15596, Invitrogen) according to the manufacturer’s protocol. The total RNA was reverse-transcribed to cDNA using the QuantiTect Rev Transcription Kits (205311, Qiagen, USA). The number of cDNA molecules in the reverse-transcribed samples was determined by real-time PCR analysis using a modified method with QuantiTect SYBR Green PCR Kits (204143, Qiagen) on an Mx3000P Real-Time PCR system (Stratagene, USA)^52^. Primers with the following sequences were obtained from BGI (BGI, China): RUNX2, 5’-TTCAACGATCTGAGATTTGTG G-3’ and 5’- GGATGAGGAATGCGCCCTA-3’; ALP, 5’-CCAACTCTTTTGTGCCAGAGA-3’ and 5’-GGCTACATTGGTGTTGAGC TTTT-3’; RANKL, 5’-CAGCATCGCTCTGTTCCTGTA-3’ and 5’-CTGCGTTTTC ATGGAGTCTCA-3’; OPG, 5’-ACCCAGAAACTGGTCATCAGC-3’ and 5’-CTGC AATACACACACTCATCACT-3’; and GAPDH, 5’-AGGTCGGTGTGAACGGATTT G-3’ and 5’-TGTAGACCATGTAGTTGAGGTCA-3’. The PCR reactions included 12.5 µl of SYBR Green I master mix, 0.25 µM of each 5’ and 3’ primer, 2 µl of sample cDNA and H_2_O to a final volume of 25 µl. A melting curve was obtained at the end of each run to discriminate specific from nonspecific cDNA products. The cDNA content was normalized by subtracting the cycle numbers of GAPDH from those of the target gene (ΔCt = Ct of target gene-Ct of GAPDH), and gene expression levels were calculated using the 2^–(ΔCt)^ method.

### Western blotting analysis

Total proteins from the long bones or cells were analyzed by western blotting using a modified method^21^. Briefly, the bone powder and cells were lysed with RIPA buffer supplemented with 1% protease inhibitor cocktail (P0013B; Beyotime) for 30 min on ice. Insoluble materials were removed by centrifuging at 12,000 × *g* for 10 min, and the supernatants were collected. The protein amount was then quantified with the BCA protein assay (P0012; Beyotime) using BSA as a standard. The sample protein was denatured in boiling water for 5 min in SDS-PAGE sample loading buffer (P0015, Beyotime). Aliquots of the samples (40 µg) were then subjected to SDS-PAGE on 12% gels under reducing conditions and electroblotted onto PVDF membranes (Ipvh00010; Millipore, USA). The membranes were blocked with 5% fat-free dry milk in TBST (0.1% Tween-20 and 0.1 M NaCl in 0.1 M Tris-HCl, pH 7.5) for 2 h at room temperature and then incubated with primary antibodies at 4 °C overnight. The antibodies include anti-BK (APC-107, Alomone Labs, Israel), anti-Runx2 (ab76956, Abcam, USA), anti-integrin β1 (1798-1, Epitomics), anti-FAK (12636-1-AP, Proteintech, USA), anti-FAK-Y397 (EP2016Y, Abcam), anti-Phospho-p44/42 MAPK (ERK1/2) (4730, Cell Signaling Technology, USA), anti-p44/42 MAPK (ERK1/2) (9102, Cell Signaling Technology), anti-Osterix (ab209484, Abcam), anti-ALP (AF1030, Beytime), anti-Flag (1:200, Rabbit Monoclonal Anti-Flag antibody, F2555, Sigma), anti-myc (Santa Cruz Biotechnology, USA), anti-his (2365, Cell Signaling Technology), anti-β-actin (ab8226, Abcam), and anti-GAPDH (ab9485, Abcam). The membranes were then incubated with horseradish peroxidase-conjugated secondary antibody (1:5000; Santa Cruz Biotechnology) at room temperature for 1 h, followed by chemiluminescence detection (P0018, Beyotime). Each incubation step was followed by three washes (10 min each) with TBST. The protein bands were quantitatively analyzed by using an image analysis system (Quantity One software; BioRad ChemiDoc, BioRad, USA).

### Co-Immunoprecipitation (Co-IP)

The interaction of BK with integrins was analyzed by Co-IP. The cells were lysed with RIPA buffer as described above. One portion of the supernatant was denatured in boiling water in SDS-PAGE sample loading buffer for whole cell lysates (input). The other portion of the supernatant (~1 mg protein in 500–800 μl) was incubated with an IP antibody (10 μl, ~2-5 μg) (anti-BK, anti-myc antibody, anti-Flag antibody or anti-His antibody) for 1 h at 4 °C in a rolling incubator. Protein A/G agarose beads (20–40 µl, sc-2003, Santa Cruz Biotechnology) were then added and incubated overnight at 4 °C in a rolling incubator. The immunoprecipitates (beads) were collected by centrifugation at 1000 × *g* for 5 min at 4 °C. The pelleted beads were washed three times with pre-cold RIPA buffer (1000 × *g* for 5 min at 4 °C) and eluted in boiling water for 5 min in 30–50 µl 2× loading buffer. The bound proteins were detected by western blot analysis.

### Statistical analysis

Data are presented as means ± SD and repeated at least in three independent experiments. Statistical comparisons were carried out using one-way ANOVA (>2 groups) or unpaired two-tail Student’s *t*-test (two group), and *P*-value < 0.05 was considered significant.

## Supplementary information

supplemental information

supplemental Figure 1

supplemental Figure 2

supplemental Figure 3

supplemental Figure 4

supplemental Figure 5
