# Supplementary material for: BK ablation attenuates osteoblast bone formation via integrin pathway
Source: Cell Death Dis. 2019 Sep 30;10(10):738. doi: 10.1038/s41419-019-1972-8 (PMC6769012; doi:10.1038/s41419-019-1972-8)
Supplement: Supplementary file 1 — supplemental information [file 41419_2019_1972_MOESM1_ESM.docx]

**Supplementary information**

**BK ablation attenuates osteoblast bone formation via integrin pathway**

Yinhang Wang, Qiang Guo, Hongya Hei, Jie Tao, Yi Zhou, Jibin Dong, Hong Xin, Hui Cai, Jianjun Gao, Peihao Yin and Xuemei Zhang

**Inventory**

**Supplementary Materials and Methods**

**Supplementary Figure Legends 1-5**

**Supplementary Figures 1-5**

**Supplementary Materials and Methods**

**Construction of osteoporosis model**

Eight female Sprague-Dawley rats (Shanghai Lab Animal Resource Center, STCSM, Shanghai, China) were randomly divided into two groups. The bilateral ovariectomies for OVX or sham surgery for SHAM group were performed respectively under anesthesia of pentobarbital (10g/L) at 1 month old. All rats were maintained in a virus- and parasite-free barrier facility and exposed to a 12-h/12-h light/dark cycle. After 12 weeks tissues were collected at the time of sacrifice and left femur were isolated and powdered in liquid nitrogen for western blot and real-time RT-PCR analyses. All experiments involving animals were performed according to institutionally approved and current animal care guidelines.

**Cell culture**

MC3T3-E1 cells were cultured in α-minimum essential medium (Gibco, USA) supplemented with 10% FBS, 100 U/ml penicillin and 100 mg/ml streptomycin. MG63 cells were cultured in Dulbecco’s Modified Eagle Medium (Gibco, USA) supplemented with 10% FBS, 100 U/ml penicillin and 100 mg/ml streptomycin. The cells were cultured in a humidified atmosphere of 95% air and 5% CO2 at 37°C and subcultured every 2-3 days. For the experiments, the cells were trypsinized at approximately 90% confluence and seeded onto 6-well plates at a density of 2.5×10^4^/ml.

**RNA interference**

Three shRNA plasmids targeted to KCNMA1 (Y4061, targeted to GCTCCTGATGATAGCCATT, Gene ID：NM_010610) were obtained from Obio Technology (Shanghai) Co. Ltd, China. The plasmid Y007 targeted to TTCTCCGAACGTGTCACGT was used as a negative control. The shRNA plasmids were transfected into ROS 17/2.8 cells in 6-well plates using Lipofectamine 3000 (L3000-015, Invitrogen, USA) according to the manufacturer’s protocol. Four μg/ml puromycin was added for selection on the second day of transfection. Two days later, the cell proteins were extracted for analysis by western blotting.

**BK overexpression**

The BKα-myc plasmid was transfected into ROS 17/2.8, MG63 and MC3T3-E1 cells in 6-well plates using Lipofectamine 3000. The MC3T3-E1 cells medium was refreshed 24 h later, and 10 μM ERK1/2 inhibitor U0126 (S1102, Selleck, USA) or 5 μM FAK inhibitor PF-562271 (S2890, Selleck) was added as needed. The cells were collected for protein analysis 2 days later.

**Whole-cell patch clamp**

Whole cell patch clamp recordings were conducted using an Axon Multiclamp 700B (Molecular Devices, USA) ampliﬁer at RT (room temperature). Patch pipettes were fabricated from glass capillary tubes by P97 (Sutter, USA) or PC-10 Puller (Narishige, Japan) with the resistance of 2-5 MΩ. Data acquisition and stimulation protocols were controlled by pCLAMP 10 (Molecular Devices, USA). Capacitance transients were cancelled. Cells with a seal resistance (Rseal) below 1 GΩ were omitted. Series resistance (Rs) was compensated to 80-85% in order to minimize the voltage errors. Cells with uncompensated Rs above 10 MΩ were discarded. During whole-cell recording, control solutions or drugs were puffed locally to the test cell by a puffer pipette containing 4 solution channels (ALA Scientific, USA). The tip, which was 300 μm diameter, of the puffer pipette was located about 120 μm from the target cell. The entire patch clamping experiments met this criterion. Outward currents of osteoblast were elicited by the step pulses ranging from -50 to +120 mV for 200 ms with the increments of 10 mV. The holding potentials were held at -80 mV. The currents evoked using these protocols are entirely attributable to BK channels, which could also be identified by exerting paxilline.

The standard external solution for BK channels contained (in mM): NaCl 150, KCl 5.4, MgCl_2_ 0.8, CaCl_2_ 5.4, HEPES 10, the pH of the solution was adjusted to 7.4 with NaOH. Internal solutions contained (in mM): NaCl 10, KCl 125, Mg Cl_2_ 6.2, HEPES 10 and 5 mM free Ca^2+^, the pH of the solution was adjusted to 7.2 with KOH. The total Ca^2+^ was added to give the desired free concentration, which was calculated using the program Maxchelator^[4, 5]^..

The raw data was collected by Origin 8.5 (OriginLab, USA). Results of data were expressed as means ± SEM and statistically analyzed by one-way ANOVA. Error probabilities of P<0.05 were considered statistically significant.

**Supplementary Figure legends**

**Supplementary Figure 1. Decrease of BK expression in bone of ovariectomized rats.**

(A) Western blot analysis of BK protein in femur of ovariectomized rats and sham group; (B) Quantitative analysis of BK protein expression level by western blot of (A). Values are shown as the mean ± SD, n=4, versus sham: **P < 0.01. (C) BK mRNA levels in femur of OVX and Sham rats. Values are shown as the mean ± SD, n=4, versus sham: **P < 0.01.

**Supplementary Figure 2. Identification of the endogenous BK channels in WT but not in BKO mice determined by whole-cell patch clamp.**

(A) Representative traces of whole cell currents from WT and (B) BKO mice with or without of 10 μM paxilline (PAX). The holding potential was -80 mV and the outward currents were evoked by the step pulses ranging from -50 to +120 mV for 200 ms with the increments of 10 mV and 5 μM free Ca^2+^ in the pipette solution. (C) Quantification of current densities at each step pulse. Compared with WT, * P < 0.05, ** P < 0.01, *** P < 0.001 by one-way ANOVA.

**Supplementary Figure 3. Expression of BK, p-FAK-Y397, p-ERK1/2, Runx2, ALP and Osterix in BK modified MC3T3-E1 cells.**

(A) Expression of BK, p-FAK-Y397, p-ERK1/2, Runx2, ALP and osterix levels in MC3T3-E1 cells after BK-shRNA transfection by western blot analysis. (B) Quantitative analysis of the protein expression levels by western blot of (A). Values are shown as the mean ± SD, n=3, versus Ctrl: **P < 0.01.

**Supplementary Figure 4. Overexpression of BK increases Runx2 through FAK and ERK in MC3T3-E1 cells.**

(A) Western blot analysis of BK, p-FAK-Y397, Runx2, ALP and osterix after the BKα-myc plasmid was transfected into MC3T3-E1 cells using the FAK inhibitor PF562271 (5 μM). PF was short for PF562271. (B) Western blot analysis of BK, p-ERK1/2, Runx2, ALP and osterix in BKα-myc transfected cells using the ERK inhibitor U0126 (10 μM). (C) Quantitative analysis of the protein expression levels by western blot of (A). Values are shown as the mean ± SD, n=3, versus Ctrl - : *P < 0.05, versus PF - : #P < 0.05, ##P < 0.01. (D) Quantitative analysis of the protein expression levels by western blot of (B). Values are shown as the mean ± SD, n=3, versus Ctrl - : *P < 0.05, **P < 0.01, versus PF - : #P < 0.05, ##P < 0.01.

**Supplementary Figure 5. Expression of BK in integrin β1 modified osteoblasts.**

(A) Western blot analysis of integrin β1 after BK-shRNA plasmid (Y2255) was transfected into MG63 cells. Reduced integrin β1 in protein level was shown in BK silenced MG63 cells; (B) Western blot analysis of integrin β1 after BKα-myc plasmid was transfected into MG63 cells. Increase of integrin β1was shown. (C) Western blot analysis of integrin β1 after ITGB1-shRNA plasmids (Y2652, Y2653) transfection in ROS17/2.8 cells. The expression of integrin β1 was knocked down by Y2653 transfection. (D) Western blot analysis of BKα in the silenced cells, which shown that down-regulation of integrin β1 did not affect the expression of BK.
