# Supplementary figures and images for: BK ablation attenuates osteoblast bone formation via integrin pathway
Source: Cell Death Dis. 2019 Sep 30;10(10):738. doi: 10.1038/s41419-019-1972-8 (PMC6769012; doi:10.1038/s41419-019-1972-8)

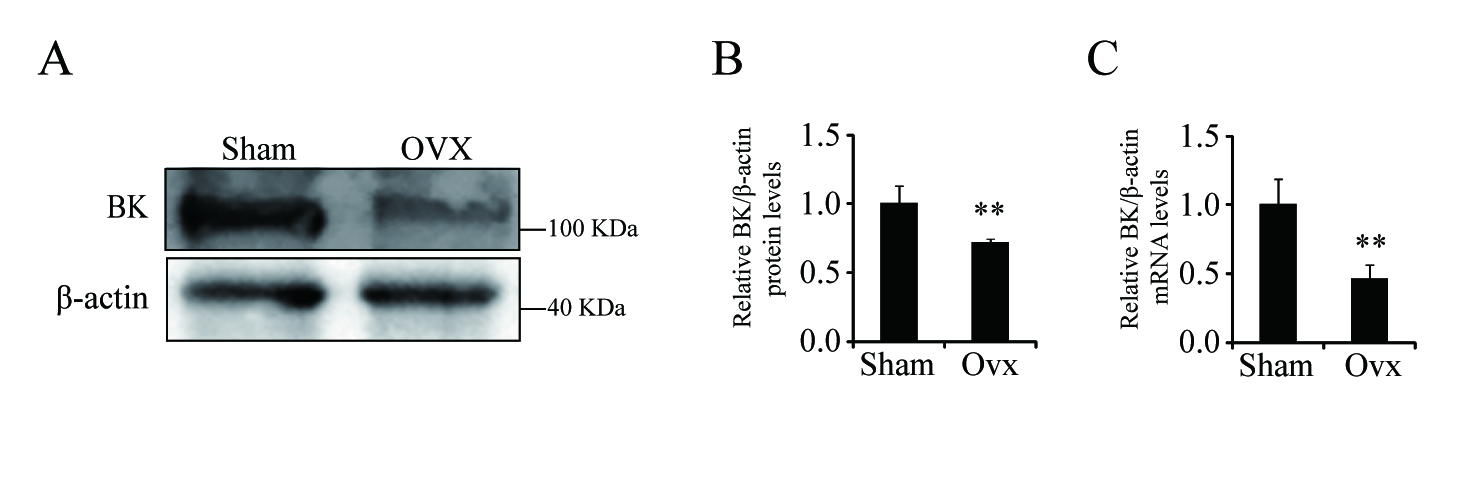

Supplement: Supplementary file 2 — supplemental Figure 1 [file 41419_2019_1972_MOESM2_ESM.tif]

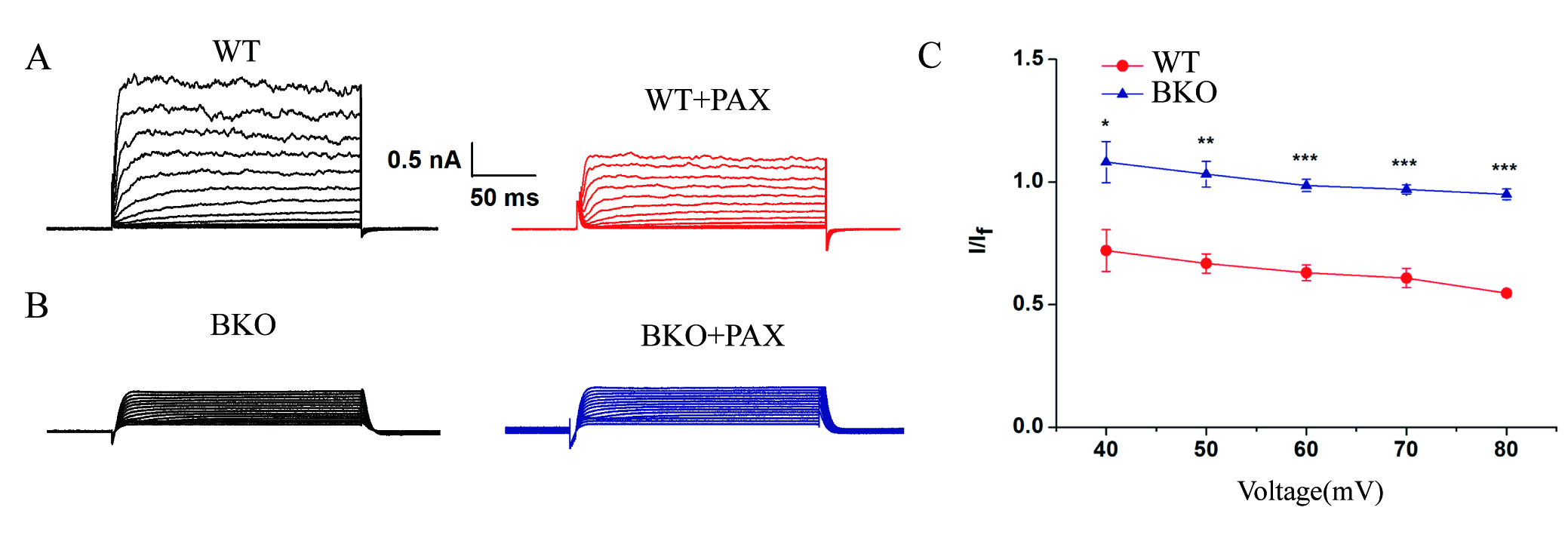

Supplement: Supplementary file 3 — supplemental Figure 2 [file 41419_2019_1972_MOESM3_ESM.tif]

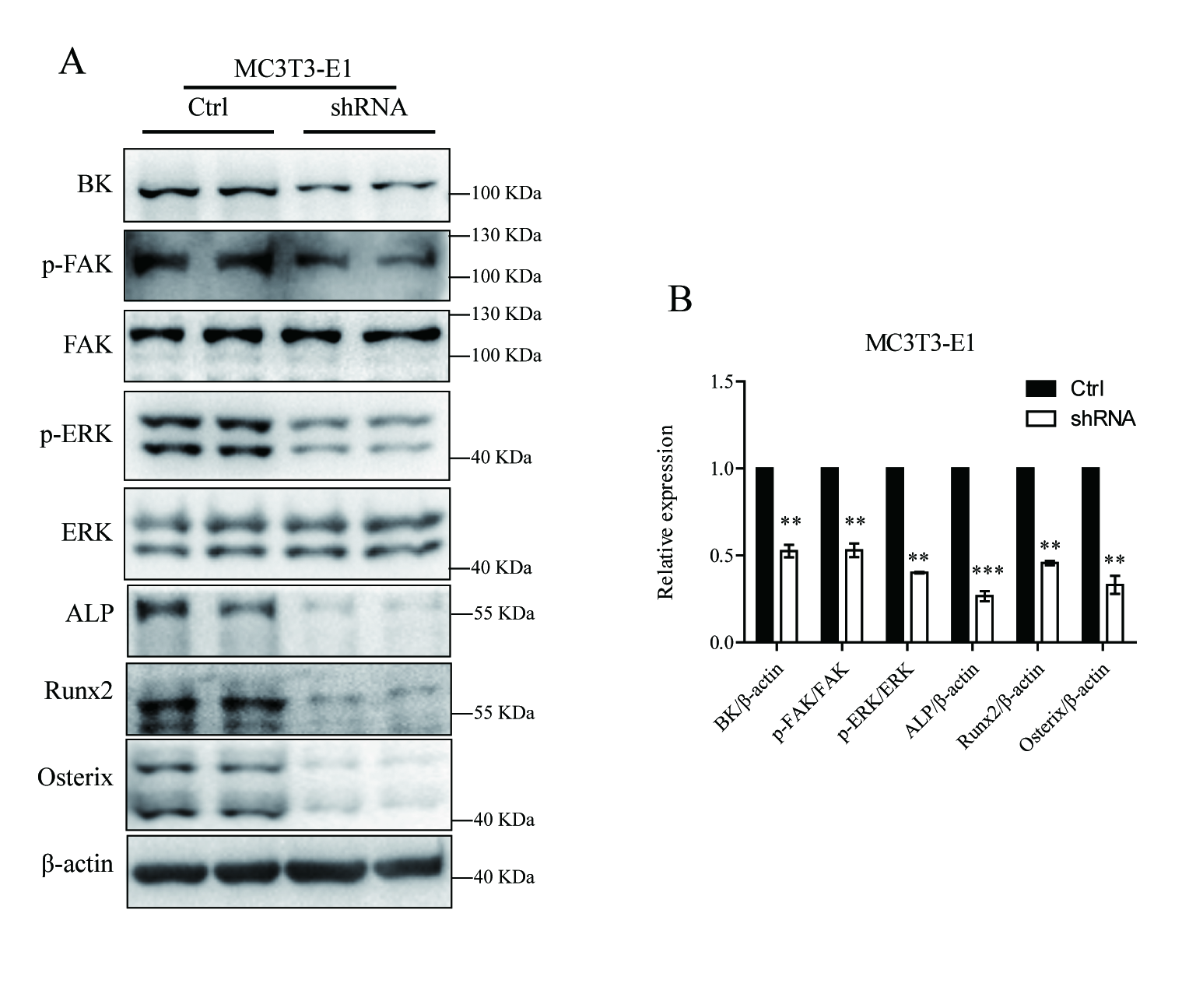

Supplement: Supplementary file 4 — supplemental Figure 3 [file 41419_2019_1972_MOESM4_ESM.tif]

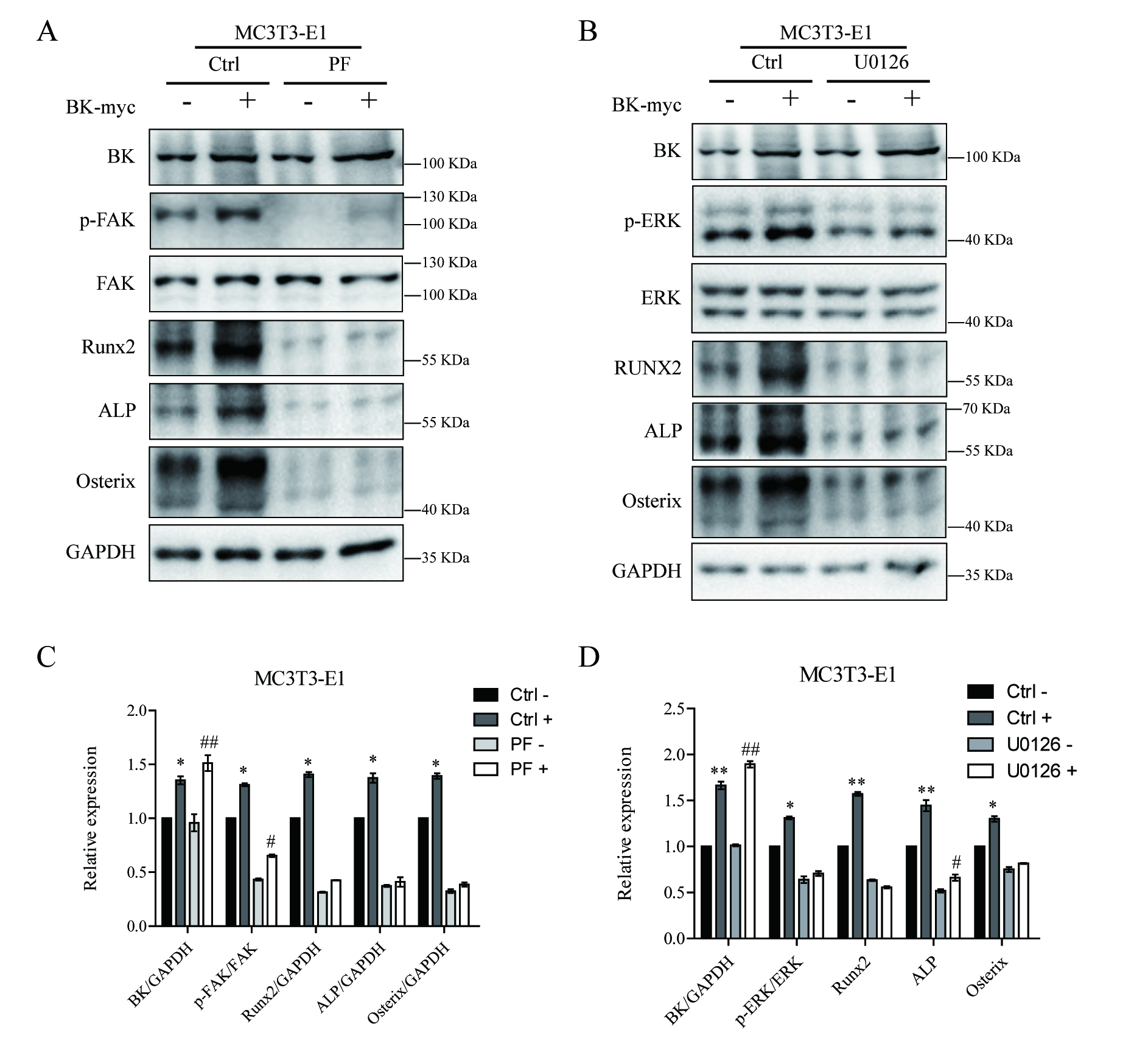

Supplement: Supplementary file 5 — supplemental Figure 4 [file 41419_2019_1972_MOESM5_ESM.tif]

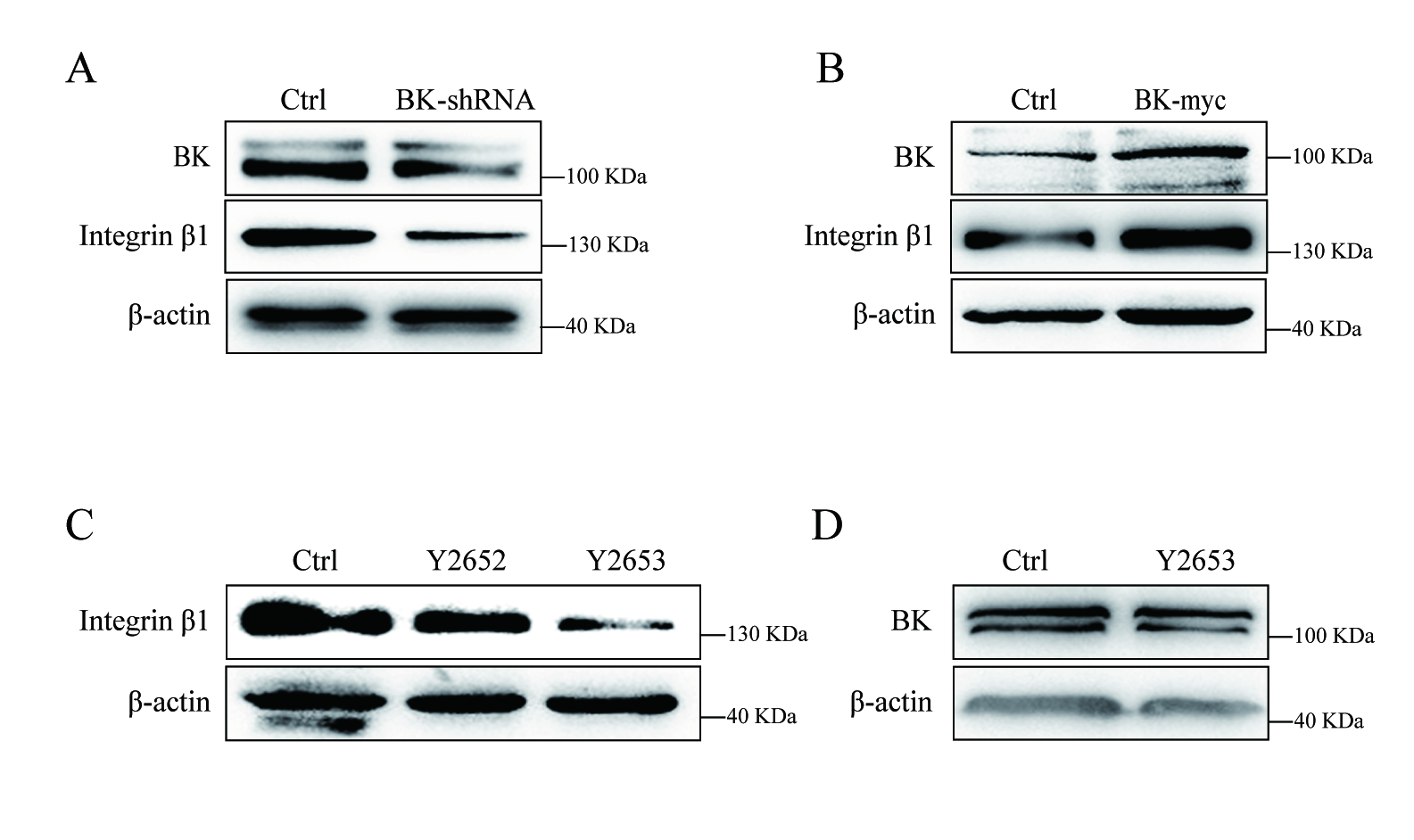

Supplement: Supplementary file 6 — supplemental Figure 5 [file 41419_2019_1972_MOESM6_ESM.tif]
